# The macrocycle inhibitor landscape of SLC‐transporter

**DOI:** 10.1002/minf.202300287

**Published:** 2024-03-05

**Authors:** Nejra Granulo, Sergey Sosnin, Daniela Digles, Gerhard F. Ecker

**Affiliations:** ^1^ Department of Pharmaceutical Sciences University of Vienna Josef Holaubek Platz 2 1090 Vienna Austria; ^2^ Research Platform NeGeMac–Next Generation Macrocycles to Address Challenging Protein Interfaces University of Vienna 1090 Vienna Austria

## Abstract

In the past years the interest in Solute Carrier Transporters (SLC) has increased due to their potential as drug targets. At the same time, macrocycles demonstrated promising activities as therapeutic agents. However, the overall macrocycle/SLC‐transporter interaction landscape has not been fully revealed yet. In this study, we present a statistical analysis of macrocycles with measured activity against SLC‐transporter. Using a data mining pipeline based on KNIME retrieved in total 825 bioactivity data points of macrocycles interacting with SLC‐transporter. For further analysis of the SLC inhibitor profiles we developed an interactive KNIME workflow as well as an interactive map of the chemical space coverage utilizing parametric t‐SNE models. The parametric t‐SNE models provide a good discrimination ability among several corresponding SLC subfamilies’ targets. The KNIME workflow, the dataset, and the visualization tool are freely available to the community.

## Graphical abstract

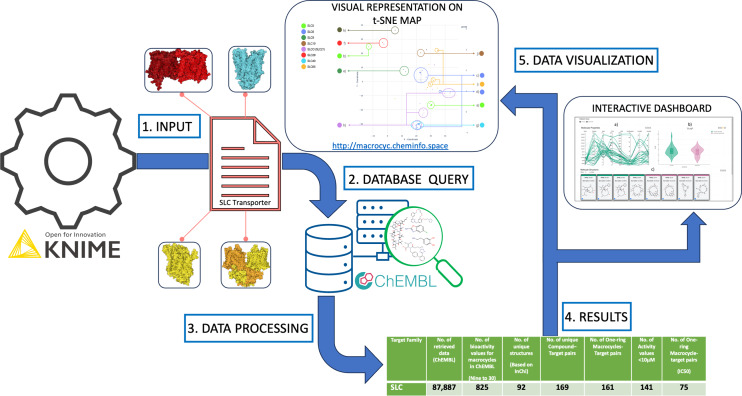

## INTRODUCTION

The Solute Carrier Transporters’ (SLC) superfamily comprises the second‐largest group of membrane proteins and the most extensive group of transporters [1]. They are grouped into 66 classical and five nonclassical families and are categorized according to the HUGO Gene Nomenclature Committee (HGNC) [2, 3, 4]. Their presence is indispensable for maintaining the vital functions of a single cell by importing essential molecules such as inorganic ions, amino acids, fatty acids, monosaccharides, neurotransmitters, and pharmaceuticals. Interestingly, despite their vital role, a considerable number of them has not been fully explored yet, and a substantial portion even needs to be deorphanized [2, 5, 6]. Considering their pivotal role in maintaining cellular homeostasis, it is not unexpected that even subtle disruptions in their functions are associated with dysregulation and disease pathogenesis. SLCs exhibit connections with an extensive spectrum of both common and rare disorders, encompassing diabetes (SLC5A2, SLC30A8), bladder cancer (SLC14A1), major depression (SLC3A1, SLC6A15), and a multitude of others, thus positioning them as an enticing cohort of drug targets [5, 6, 7]. Despite representing only a limited fraction of the number of drug‐like compounds, macrocycles have captured the interest of researchers exploring ligands for challenging drug target classes [8]. The architectural arrangement of macrocycles (defined herein as structures possessing a ring size of at least nine atoms) allows them to strike a balance between structural pre‐organization and sufficient adaptability to accommodate protein binding sites and protein–protein interfaces [9]. Despite their divergence from strict adherence to the well‐known “rule of five” [10], macrocycles demonstrate notable attributes such as favorable cell permeability, increased metabolic stability, and the capacity to effectively modulate protein‐protein interactions, all while incurring minimal entropic cost [11, 12]. To name a few milestones, the discovery of Erythromycin, for instance, has not only provided a solution for patients with penicillin allergies, but also introduced the class of macrolide antibiotics. Additionally, the remarkable effectiveness of Cyclosporine revolutionized the field of organ transplantation, transforming it from a high‐risk procedure into a routine technique. Cyclosporine's ability to selectively suppress the immune system has become a standard practice in contexts where immunosuppression is necessary [13, 14, 15]. Naturally occurring macrocycles encompass a diverse range of compounds, including peptides and nonpeptides, which have been mimicked through synthetic approaches by researchers. However, within the vast chemical landscape of small molecules, macrocycles remain relatively underexplored and undervalued [16, 17, 18, 19]. This can be observed through the number of macrocyclic drugs endorsed by the U.S. Food and Drug Administration (FDA). The FDA has given its approval to 67 macrocyclic drugs, primarily utilized for addressing infectious diseases. Notably, subsequent key applications extend to cancer therapy, immunosuppression, and the treatment of autoimmune diseases. While there are additional therapeutic applications in areas such as chronic pain, genetic obesity, and heart failure, it is important to highlight that a considerable majority of medical indications are still managed by non‐macrocyclic drugs [20]. Recognizing the inherent potential of macrocyclic properties, there is a growing initiative to introduce them as a novel modality in drug design and development. The constrained flexibility of macrocycles is a key advantage over small molecules, enabling highly specific interactions with crucial residues. This not only enhances potency but also enables the targeting of more intricate binding interfaces, leading to higher selectivity. In the context of ligand design for matrix metalloproteinases (MMPs), macrocyclization has led to the discovery of more selective compounds. Thus, comparative studies reveal that macrocyclic ligands exhibit a 17‐fold increase in activity on MMP8 compared to their linear analog, while demonstrating either reduced or unchanged activity on MMP1, MMP2, MMP3, and MMP9 [13, 20]. Recent studies conducted by Jimenez et al. demonstrate that FDA approved macrocycles are very effective in tackling flat‐, groove‐ and tunnel‐shaped binding sites, which usually pose a challenge for small molecules [20]. Transporters such as SLCs also comprise–among others–tunnel‐shaped binding pockets, opening up the possibility to develop more efficient drugs and increase the applicability area of macrocycles. Moreover, the macrocyclization of linear ligands has proven to be a valuable strategy for enhancing affinity by mitigating entropic loss. For instance, the macrocyclization of dapagliflozin, a potent inhibitor of SGLT2 (SLC5A2), resulted in increased target affinity, showcasing a novel approach towards the development of more potent SGLT2 inhibitors [13, 21]. In the context of solute carriers (SLCs), a recent in‐depth analysis of the interaction landscape between small molecule ligands and SLCs in the ChEMBL [22] database, conducted by Schlessinger et al., reveals a notable imbalance towards a small subset of SLCs. These comprise members of the GLUT family (SLC2) and the SLC6 family (SERT, DAT, NERT), for which there are more than thousand reported ligands. In contrast, a substantial majority of SLC transporters lack recorded ligands within the database [23].

Acknowledging the significance of SLC transporters for balancing the chemical and physiological processes while considering the unique potential of macrocycles in targeting complex binding sites, it could be of interest to provide an overview of the interaction landscape between SLC transporter and macrocycles.

Therefore, we have developed a KNIME workflow to provide a thorough investigation of the interaction between macrocyclic ligands and SLCs, as well as any other target classes, utilizing publicly available data sources. Visualization of chemical space by dimensionality reduction methods has gained popularity recently, and several visualization frameworks have been created, for example, ChemPlot [24]. Thus, in order to enable the visual exploration of the macrocyclic ligand landscape and its associations with specific SLC transporter families, we have created a dedicated tool. Our tool allows the visualization of a molecular set of ligands as points within a two‐dimensional (2D) plane, allowing researchers to navigate the chemical space of macrocyclic SLC‐transporter ligands. The tool's source code is freely available on GitHub (https://github.com/PharminfoVienna/macrocyc). Also, it is accessible online at http://macrocyc.pharmapp.univie.ac.at.

## METHODS

The core technique used in our study is the data retrieval and analysis workflow that can extract, collect, and analyze bioactivity data. The workflow operates as a tool for extracting data from the ChEMBL [22] database for a specific target group. The visualization of the landscape is provided as an interactive web‐based tool written in Python (http://macrocyc.pharmapp.univie.ac.at).

The following sections elaborate on the fundamental steps of the data mining approach without providing a detailed account of each node and its specific configuration. The workflow is available on the KNIME Hub and can be applied on any other target class of interest.

### Data mining from ChEMBL and determination of the ring size

The fundamental element in the “Search Part” of the workflow represents the file RESOLUTE_SLCs (xlsx format, Supporting Information) provided by “RESOLUTE” [25], which contains a list of 446 SLC transporters. The column “UniProt Accession ID” is necessary for conveying data via the “ChEMBL Target Pharmacology Node.” To mine data from the ChEMBL database, one needs to allocate either the UniProt Accession ID or the Target ChEMBL ID of the target(s) of interest to retrieve the information available in ChEMBL. The access to ChEMBL is conducted by the “ChEMBL target pharmacology” metanode, which queries the database via its API. For differentiating between macrocycles and non‐macrocycles, we employed SMILES arbitrary target specification (SMARTs) [26] to define the ring size of macrocycles, setting a range from nine to 30 [18].

The retrieval of the molecule‐target pairs is succeeded by performing a duplicate check based on InChI [27]. Herein, duplicates are defined as compounds for which more than one activity value for the same transporter was retrieved, with the same “Standard Type” and the same “Standard Value.” Additionally, we utilized the InChI to unveil the number of unique structures in the dataset.

Upon inspecting our dataset visually, we identified the presence of macrocycles featuring peptide‐like motifs. To enable a distinct analysis of macrocycles with and without peptide‐like motives, we introduced a component node that differentiates between these groups. The formation of an amide bond, resulting from the linkage of two or more amino acids, serves as a standardized pattern in our methodology. Due to the wide variety of natural products, it is challenging to accurately identify cyclopeptides and close derivatives. For the purposes of this paper, we adopt a simplified criterion for identifying macrocycles having “peptide‐like motifs”: the presence of at least three peptide bonds within the closed loop.

**Figure 1:**
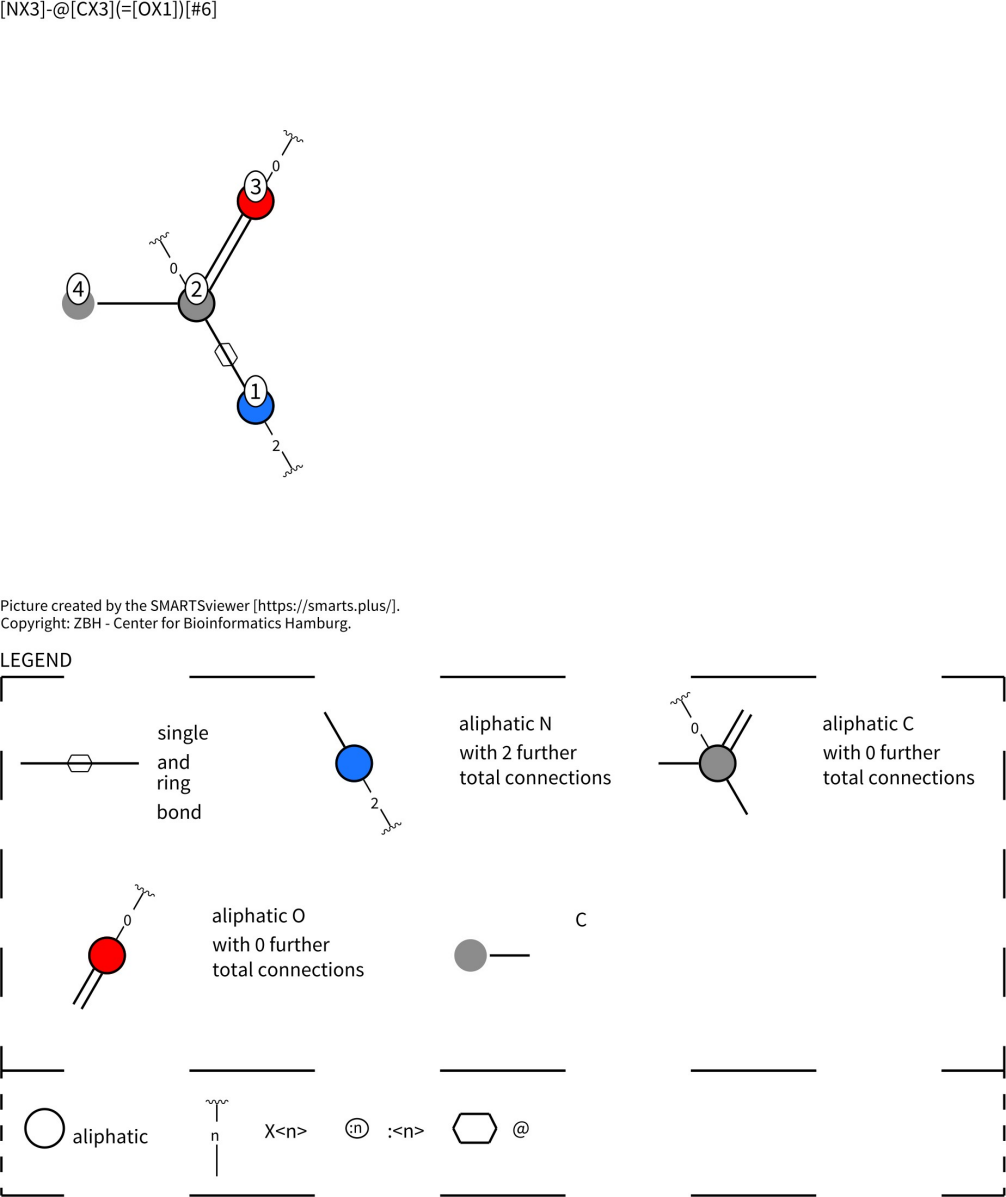
The visualization of “[NX3]‐@ [CX3] (=[OX1])‐@ [# 6]” SMARTS pattern that we used to distinguish between macrocycles with peptide‐like motives and without.

The motif used unites the two desired conditions, so “[NX3]‐@[CX3] (=[OX1])[# 6]” identifies the amide, and the “@” sign emphasizes the amides that are the building blocks of a circuit [26]. In addition to the “Table Creator” and “SMARTs query” mentioned earlier, two “RDkit Substructure Counter” nodes were also required. The visualization of this SMARTS pattern shown in Figure 1 was created by SMARTS.plus [28].

### Visual representation of molecular properties

**Figure 2:**
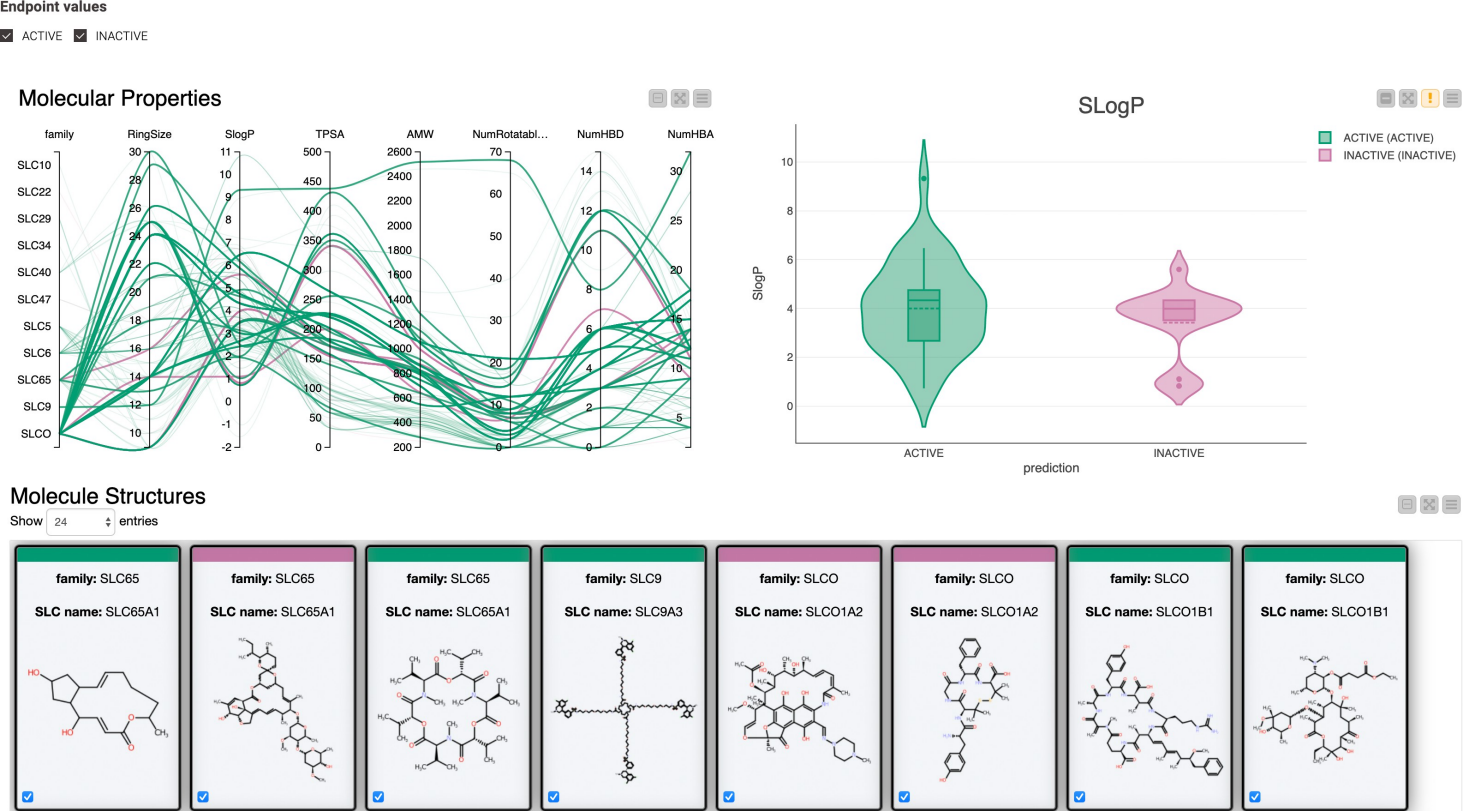
Interactive Dashboard for Visualization of Molecular Properties integrated in KNIME Workflow: The Dashboard is composed of (a) an interactive Parallel Coordinate Plot that shows the calculated physicochemical descriptors and ring size of active (colored in green) and inactive (colored in pink) macrocycles (Activity Threshold=10 μM), as well as the SLC families to which they belong to. One can move the cursor along one vertical axis or more to explore the relationship between them; (b) an interactive Violin Plot that shows the distribution of SlogP values of active and inactive compounds; and (c) an interactive Structure Tile View. All three sections are interactive, and any modification, such as sliding the cursor along a vertical axis in “Molecular Properties,” not only reveals the distribution of SLogP values in the Violin Plot but also showcases the structures of compounds in the Tile View. (Note: In the Tile View's “Molecule Structures,” it's important to note that the examples provided are just a subset of a much larger group.). For the showcase purposes, we marked the cursor on the “Family” vertical axis (SLC65, SLC9, and SLCO), accordingly the distribution of SLogP values for both active (**green**) and inactive (**pink**) compounds is visible. At the bottom, the Tile View displays their structural representation, the name of the SLC family, and the SLC transporter they interact with. The Tile View is color‐coded, using green tiles for active compounds and pink tiles for inactive compounds. Note: The user can choose to observe only “**ACTIVES**” or only **“INACTIVES**” by clicking on the checkbox next to these two terms found in the top left corner in “Molecular Properties” and in the right corner in the Violin Plot.

For interactive visualization of the data within the workflow, several elements of the “ML Prototyping for Bioactivity Data” [29] workflow presented at a KNIME Webinar were implemented and adjusted accordingly. For a visual representation of active and inactive compounds, the activity threshold was set to 10 μM. Furthermore, we calculated several molecular descriptors such as SlogP, TPSA, AMW, number of rotatable bonds, number of hydrogen bond donors, and number of hydrogen bond acceptors, using the “RDKit Descriptor Calculation” node. These features are assembled by three visualization possibilities that are part of the component “Explore Molecular Properties.” (Figure 2).

### Parametric t‐SNE

**Figure 3:**
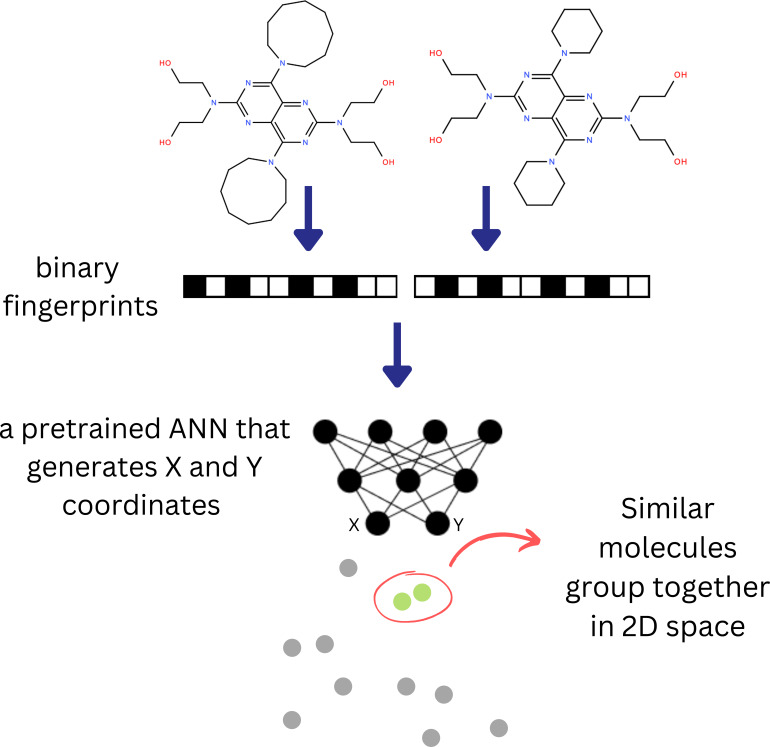
A schematic representation of parametric t‐SNE modelling. The two chemical structures of interest are converted to binary fingerprints, which serve as input for a pretrained artificial neural network (ANN). The output of the ANN are 2D coordinates which allow the positioning of the two compounds in the chemical space.

To demonstrate the structural landscape of SLC‐transporters, we used a parametric t‐SNE model from our previous research. While the detailed description of the parametric t‐SNE is beyond the scope of our study, the procedure for training this model has been described in Karlov et al. [30]. Parametric t‐SNE is a further development of the well‐known t‐SNE method [31]. Originally, the t‐SNE algorithm minimizes the Kullback–Leibler (KL) divergence between the distributions in high‐dimensional space (the space of fingerprints) and low‐dimensional space (coordinates on 2D plane) by moving the points in low‐dimensional space. In parametric t‐SNE there is no direct optimization of points, in contrast, the coefficients of an artificial neural network are optimized the same using (KL) divergence as a loss function. At the first stage, we calculates ECFP fingerprints [32] (implemented in RDKit) with the radius three and convert them into 2048‐bit vectors. Then, the pretrained feed‐forward artificial neural network is used to convert these vectors into 2D vectors representing the X and Y coordinates on a 2D plane. The main idea of the parametric t‐SNE method is that the model is trained to preserve the distances between the fingerprints (in the high‐dimensional space), making the points in 2D (the low‐dimensional space) follow the same distribution. It was shown [30] that the projections obtained by a parametric t‐SNE model are chemically reasonable because structurally similar compounds group together, forming clusters with clear chemical sense. A scheme demonstrating the application of a pre‐trained parametric t‐SNE model is given in Figure 3.

### Identifying the commonly used macrocycles

How to spot commonly used compounds such as e. g., macrolide antibiotics among all macrocycles retrieved in this study revealed to be quite challenging. One can consider the frequency of a compound's appearance in scientific literature, however, seems challenging task within an entirely automated pipeline and typically necessitates manual analysis. Here we propose an alternative approach, which utilizes the number of PubChem synonyms associated with the compounds as a measure to estimate their level of recognition. To perform such analysis, we collected all synonyms of our compounds from PubChem. To communicate with the PubChem API, we used the python library PubChemPy.

**Figure 4:**
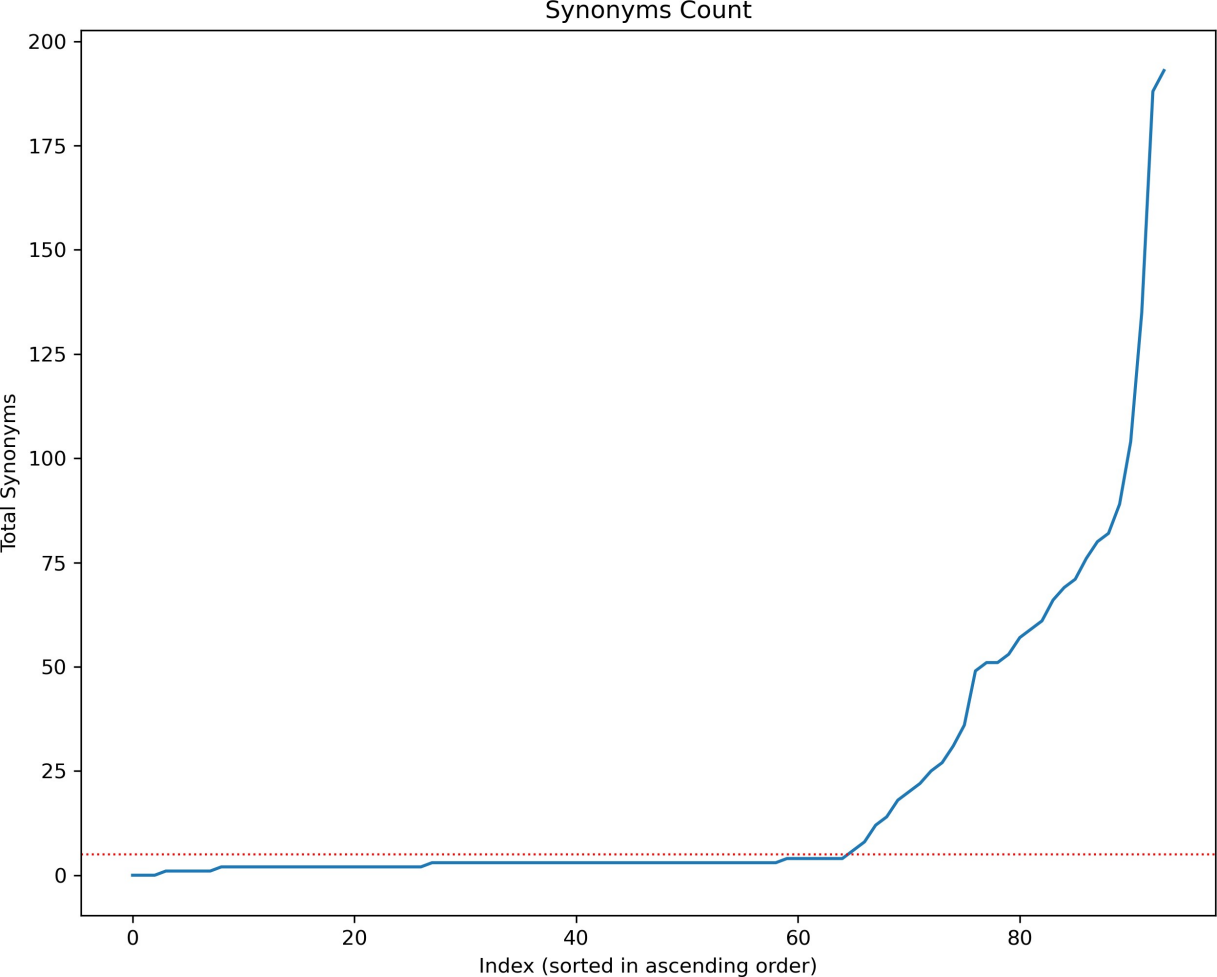
The number of total synonyms vs. the index of compounds sorted in descending order. The red line corresponds to a total number of synonyms equal to five.

To establish a threshold value distinguishing well‐recognized compounds from ordinary ones, we sorted all macrocycles in ascending order based on the count of synonyms they possess and illustrated this in a chart (Figure 4). The overlap point, marked by a red dotted line in Figure 4, indicates where there is a notable increase in the number of synonyms; this point occurs at about five synonyms. We used this threshold to differentiate between well‐recognized and ordinary compounds.

### Visualization tool

**Figure 5:**
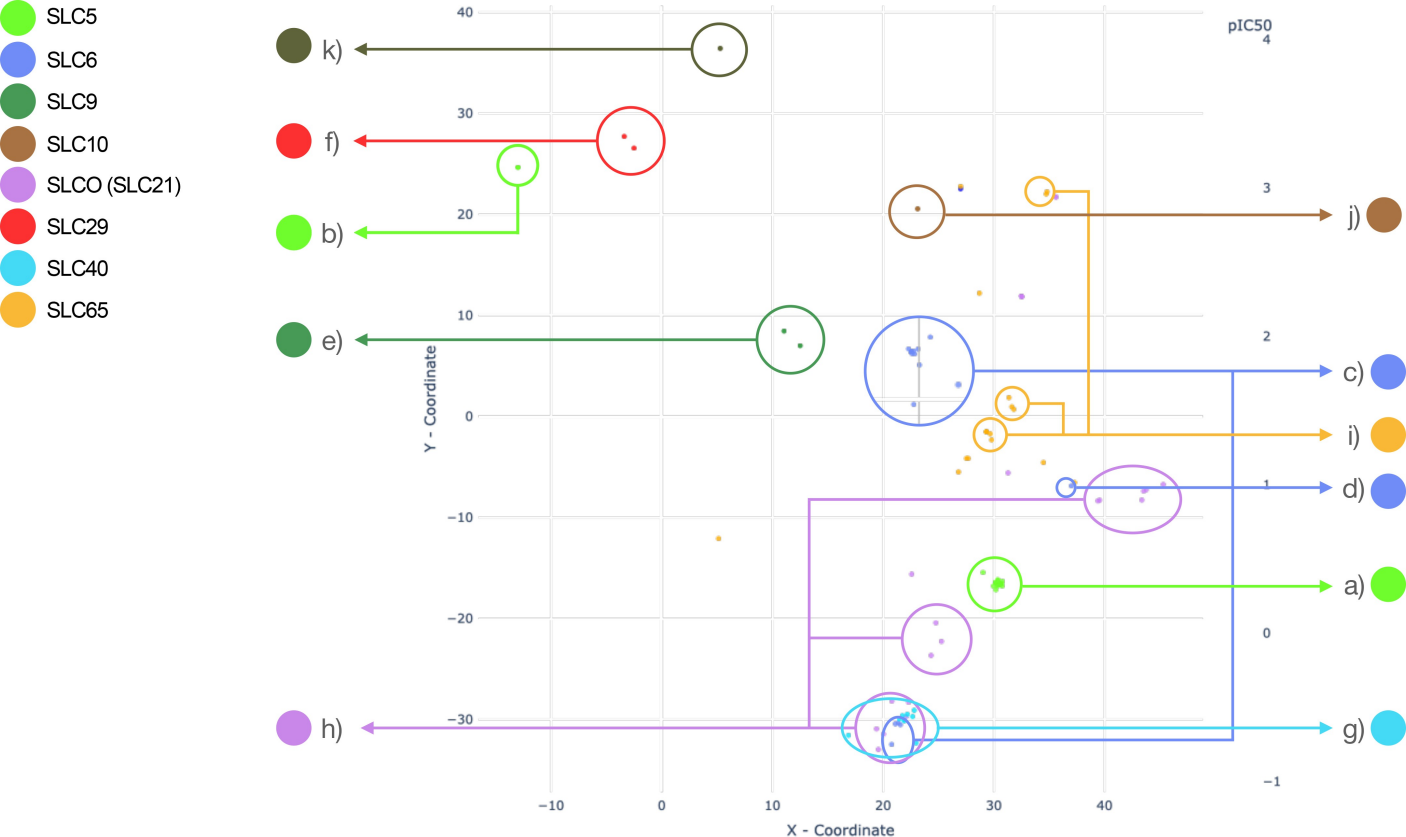
Visual representation of SLC family clusters and outliers on t‐SNE map: Every SLC family is color coded and the color of the cluster corresponds to the color of a SLC family a) SLC5; b) SLC5 outlier; c) SLC6; d) SLC6 outlier‐Ivermectin; e) SLC9; f) SLC29; g) SLC40; h) SLCO (SLC21); i) SLC65; j) SLC10.

In addition to the integrated interactive dashboard within the KNIME workflow (Figure 2), we have created a dedicated tool to provide an interactive ability to explore the chemical space of macrocyclic SLC‐transporters ligands (Figure 5). The tool is written in Python and utilizes the Plotly and Dash visualization libraries. The main area of this tool represents a scatter plot where points correspond to macrocyclic ligands. The tool provides the possibility to highlight each SLC‐family independently, zoom the map, and inspect the points and corresponding chemical structures with aggregated activity values.

## RESULTS AND DISCUSSION

The execution of the “Search part” of our pipeline retrieved 87,877 data points for SLC transporters (for ChEMBL v.30). Narrowing down the ligand space to macrocycles (ring size nine to 30) remarkably reduced the dataset to 825 data points. Hence, in the ChEMBL Database the macrocycles populate less than 1 % of ligands interacting with SLC transporters. The target space covers 31 out of 446 SLCs, belonging to 14 out of 70 SLC families. To refine our analysis and exclusively focus on macrocycles while excluding smaller cycles, we employed SMARTs to define the desired ring size range. The selected range, spanning from nine to 30, was expected to yield 22 distinct size values. Interestingly, it was observed that a macrocyclic ring composed of 27 atoms did not occur in our dataset. Notably, in addition to single cyclic frameworks, we also collected structures with two, three, or even four interconnected rings. As a result, the retrieved dataset comprised a total of 31 unique ring scaffolds. Analyzing the distribution of different ring sizes per SLCs provided valuable insights. Notably, the “Sodium‐dependent noradrenaline transporter” (SLC6A2) was associated with 22 out of the 31 identified scaffolds, highlighting its pharmacological importance for SNRIs (selective noradrenaline reuptake inhibitors). Following closely, the “Organic anion transporter” (SLC21A6) exhibited a significant association with 20 scaffolds. It is noteworthy that ten SLC transporters exclusively showed a correlation with single‐ring scaffolds, emphasizing their distinctive structural preferences within the macrocyclic landscape (Table S1, Supporting Information).

Contrarily, the analysis of SLCs per ligand ring size demonstrated a motif of 14 atoms for 18 out of 31 SLCs (58,1 %), followed by the size 15 that occurs 15 times (48,4 %), and a ring size equal to 18, which is linked to 13 SLCs (41,9 %) (Table S2, Supporting Information).

To provide a high‐quality bioactivity dataset, we filtered out data points that do not have standard nanomolar (nM) records. Furthermore, we excluded all interval records (marked as > or <) and filtered out the duplicates, which considerably reduced the number of data points to 169 compound/target pairs (out of originally 825 data points), with a total of 92 unique chemical structures.

As already stated, upon visual inspection of our dataset we noticed macrocycles having peptide‐like motives in their ring. We identified a total of 30 macrocycle–SLC pairs (approx. 25 %) where the macrocycle comprises at least 3 peptide bonds. Nine out of these 30 macrocycles associate with SLC40A1, 16 with members of the SLC21 family (SLC21A1, SLC21A6 and SLC21A8), four with members of the SLC6 family (SLC6A2 and SLC6A3), and one with SLC65A1. Consequently, the remaining 139 data points do not have peptide‐like motives in their macrocyclic ring.

### One‐ring macrocycles

As previously indicated, the dataset encompasses not only one‐ring macrocycles, but also two‐ring, three‐ring, and four‐ring macrocycles. However, the final dataset displayed limited representation of multi‐ring macrocycles. Specifically, we observed a total of six two‐ring macrocycle/target pairs, one three‐ring macrocycle/target pair, and one four‐ring macrocycle/target pair. Consequently, in order to conduct a more comprehensive analysis, our attention was primarily directed towards the investigation of one‐ring macrocycles, encompassing both macrocycles with peptide‐motives and others.

Since in high throughput screening the activity value of 10 μM is often regarded as active/inactive threshold [33], we filter out data points having activity values above 10 μM (10,000 nM), which led to a reduction to 141 data points.

One of the curated information about SLC‐macrocycles pairs is the recorded “Activity type”. In the column “Standard Activity Type” in ChEMBL one can find if the recorded values in columns “Standard Value” or “pChEMBL Value” correspond to IC50, EC50, Ki, Km, or Potency. In our dataset there are 75 IC50 values, 14 EC50 values, 35 Ki values, 15 Potency values and two Km values. However, to be able to compare recorded values we decided to use the pChembl_values. By the definition, a pChembl value is described as −log10 (molar IC50, XC50, EC50, AC50, Ki, Kd or Potency) [34].

For the 75 SLC‐macrocycle pairs with activity values recorded as IC50, pIC50 values range from 5.03–9.72 Parametric t‐SNE projection (Figure 5) reveals that there are well‐shaped clusters for most SLC‐transporters, which indicates similar underlying scaffolds for the structures.

**Figure 6:**
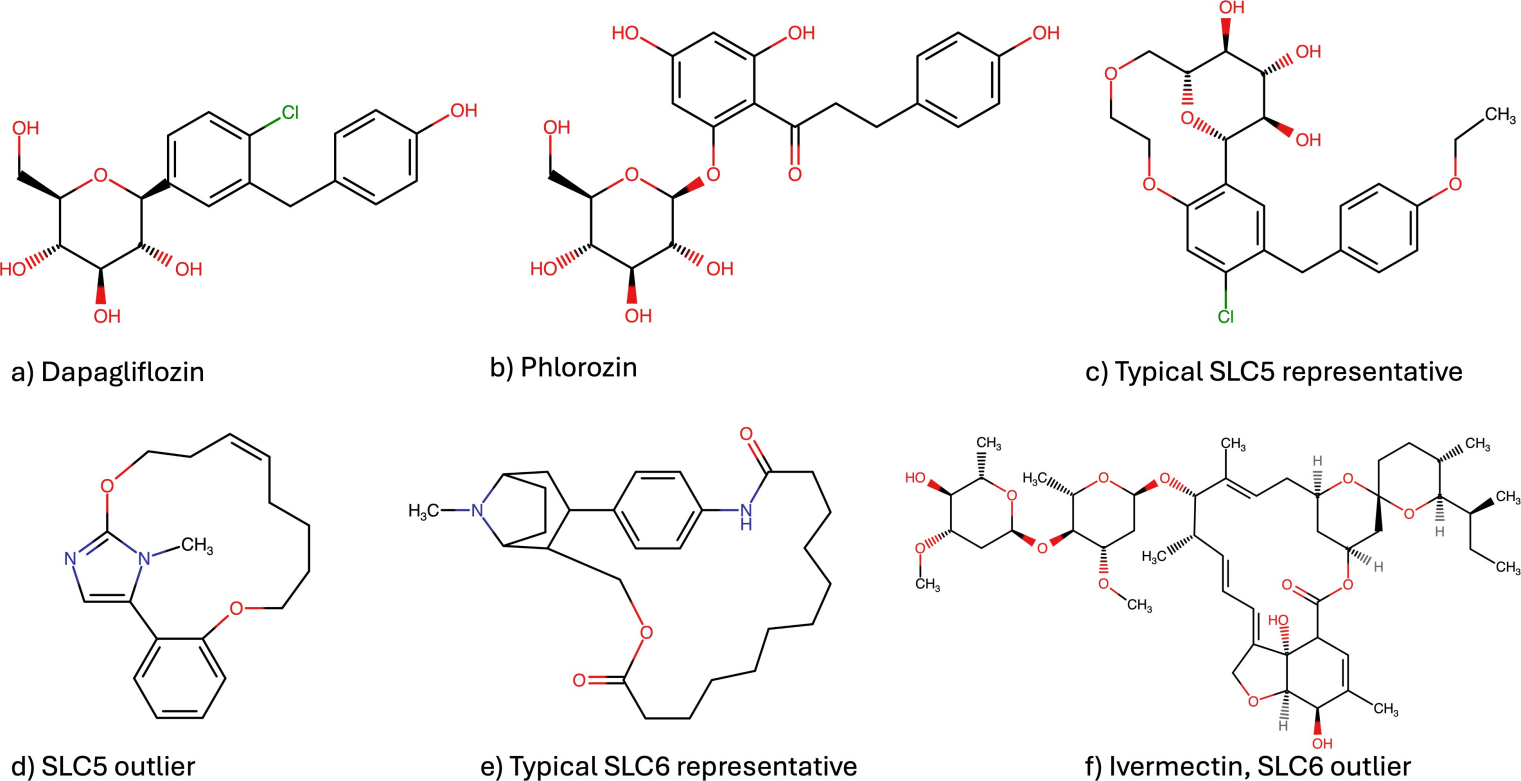
Established drugs (a) and natural products (b) for the SLC5 family as well as a typical macrocyclic representative (c) and one structural outlier (d). Structures (e) and (f) show two macrocycles interacting with SLC6 transporter.

The ligands targeting the SLC5‐transporter family known as sodium‐dependent glucose transporters, form a densely populated and highly distinct cluster in our dataset (Figure 5a), represented by a vibrant green color. The SLC5 family consists of 12 members but this cluster primarily consists of compounds that interact with three SLC5 transporters: SLC5A2/SGLT2 (Sodium/glucose cotransporter 2), SLC5A4/SGLT3 (Low affinity sodium‐glucose cotransporter), and SLC5A7/CHT (Choline transporter). Based on the substrate selectivity within the SLC5 family, SLC5A2 and SLC5A4 are classified under the group responsible for sugar transport, particularly glucose transport. On the other hand, SLC5A7, which is responsible for choline transport, appears to be more distantly related to other members of the SLC5 family in terms of substrate specificity [35]. The scaffold reveals resemblance to the drug class “Gliflozins”, also known as SGLT2 inhibitors used in antidiabetic therapy. Indeed, the scaffold of macrocycles in this cluster looks like Dapagliflozin (Figure 6a) with an alkyleneoxy group bridging the two aromatic rings [21]. Gliflozins trace their origins to Phlorizin (Figure 6b), a natural product derived from the root bark of apple trees [36, 37]. However, due to poor bioavailability, instability, and the dual inhibitory effects on both SGLT2 and SGLT1, there has been a pursuit to design more selective SGLT2 inhibitors. This endeavor has resulted in the development of a distinct drug class targeting Diabetes Mellitus Type 2 and, additionally, Heart Failure (HF) [37, 38, 39]. Most of the compounds in this cluster have high potency, with pIC50 values from 6.99 to 9.05. Notably, there is one compound far away from this cluster, showing an pIC50 value of 5.5 (Figure 5b, and 6d).

The macrocycle depicted in Figure 6d exhibits a distinct structural motif characterized by the fusion of a methylimidazole moiety with a macrocyclic ring. In contrast to the prevalent pentose motif observed in the main cluster (Figure 6c), this scaffold demonstrates considerable deviations in its chemical composition. Notably, the structural features of this macrocycle bear resemblance to 5‐Aryl‐1H‐imidazole derivatives, a class of compounds known for their diverse biological and pharmacological activities. 5‐Aryl‐1H‐imidazoles have been reported to possess significant antitubulin and antiproliferative properties, highlighting their potential in the context of antitumor therapy [40, 41].

This is a good example demonstrating how the parametric t‐SNE method can be used to quickly detect scaffold outliers.

The SLC6 family encompasses a diverse set of 20 transporters that play critical roles in the transport of various substances, including amino acids, monoamines, gamma‐aminobutyric acid (GABA), as well as creatine, taurine, and betaine [42]. Although the SLC6 family encompasses various transporters with distinct substrate specificities, our dataset specifically focuses on inhibitors targeting the monoamine transporter (MAT) subgroup. The MAT subgroup consists of three prominent transporters: SLC6A2 (norepinephrine transporter, NET), SLC6A3 (dopamine transporter, DAT), and SLC6A4 (serotonin transporter, SERT). These transporters have garnered interest due to their involvement in modulating various signaling pathways in the brain, rendering them compelling and important drug targets [43, 44]. The SLC6 (Neurotransmitter transporter family) inhibitors (depicted in the cornflower blue cluster) fall into two well‐discriminated groups (Figure 5c), along with one singleton molecule (Figure 5d). The first group is composed of analogues of cyclo‐3β‐(4‐aminophenyl)‐2β‐tropanemethanol (Figure 6e) [45]. The recorded pIC_50_ values range from 5.13–8.42. But for most compounds, the activity is moderate. The singleton is Ivermectin (Figure 5d and 6f), an antiparasitic drug [46]. It is a macrocyclic lactone, and although it shares structural similarities with macrolide antibiotics it does not possess antibiotic activity. The second cluster in the SLC6 family will be discussed in the section: “Two‐, three‐ and four‐ring macrocycles”.

In our dataset we further observe two distinct pairs of compounds: inhibitors of the SLC9 family (Sodium/Hydrogen Transporter Family) forming the emerald‐green cluster, and inhibitors of the SLC29 family (Facilitative nucleoside transporter family) forming the red cluster (Figure 5e,f, respectively). While both groups of compounds exhibit macrocyclic fragments, their overall structural motifs are more intricate and diverse.

The SLC9 family comprises a total of 13 transporters, which play vital roles in maintaining cellular homeostasis. Although these transporters exhibit diverse functions, our dataset specifically focuses on inhibitors targeting SLC9A3, also known as sodium/hydrogen exchanger 3 (NHE3). This transporter is predominantly expressed in the intestine and kidney. Dysregulation in the function of SLC9A3 has been implicated in various disorders, including Secretory Sodium Diarrhea and Cystic Fibrosis [47, 48, 49, 50].

For instance, the two inhibitors in our data set targeting SLC9A3 possess complex molecular structures characterized by a central cyclen (1,4,7,10‐tetrazacyclododecane) core. These compounds feature four functional groups connected to the core via N‐[2‐[2‐[2‐(2‐aminoethoxy) ethoxy] ethoxy] ethyl] acetamide linkers (refer to Figure S1, Supporting Information). It is noteworthy that the inhibitory properties of these compounds are likely attributed to the functional groups, such as 3‐[(4S)‐6,8‐dichloro‐2‐methyl‐3,4‐dihydro‐1H‐isoquinolin‐4‐yl] benzenesulfonamide, rather than the macrocyclic core itself. These SLC9A3 inhibitors have been previously described in US Patent No. US10272079B2 [51], where they were identified as potential inhibitors of phosphate transport. This suggests their potential as therapeutic candidates targeting SLC9A3 and related pathways involved in cellular homeostasis.

**Figure 7:**
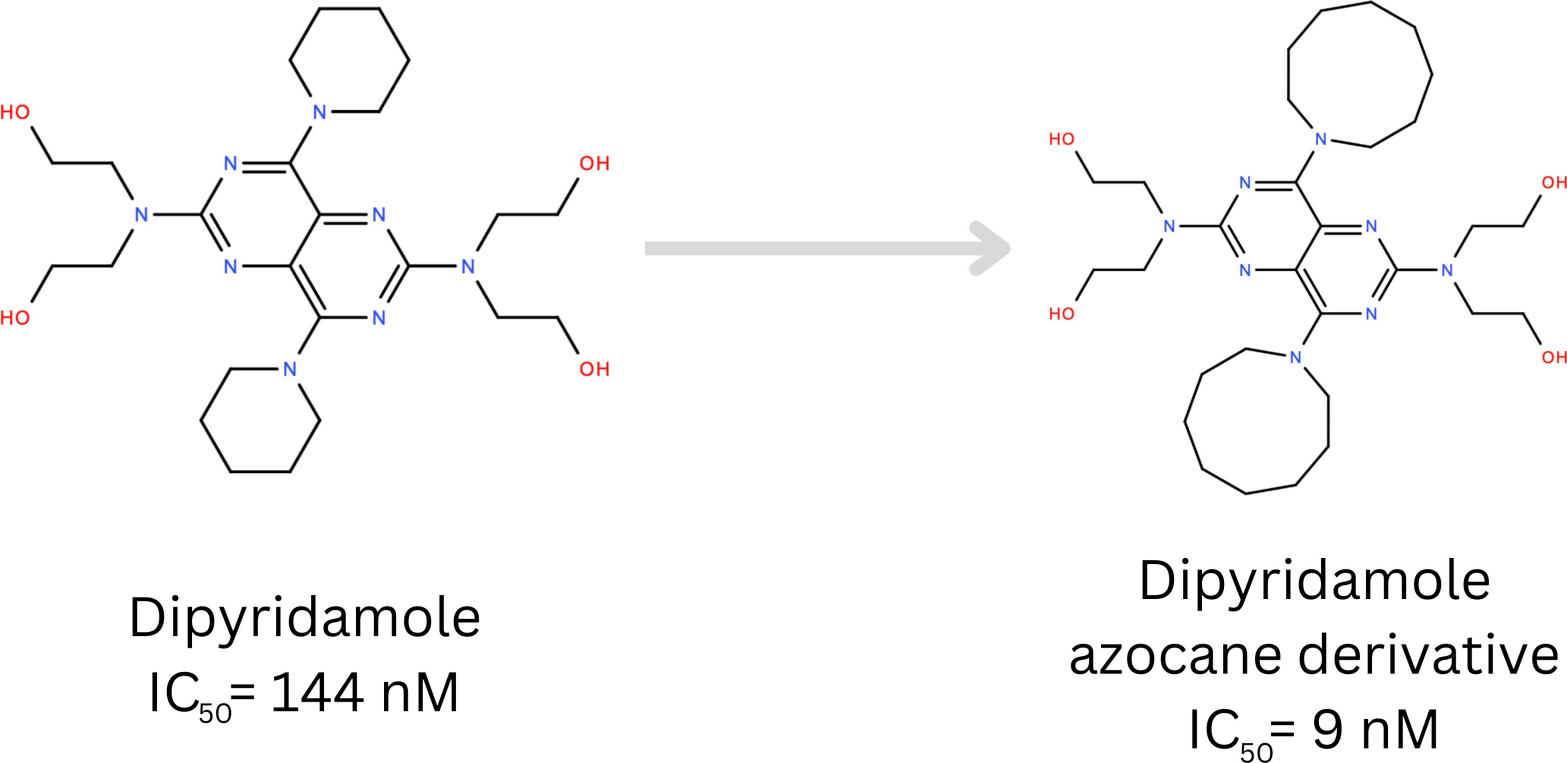
Modification of the dipyridamole structure by, introducing an azocane moiety led to improved bioactivity; From Lin et al. [52].

Another interesting example can be found in the inhibitors of the SLC29 family (red cluster). These inhibitors share a common 4,8‐bis(azonan‐1‐yl) pyrimido[5,4‐d] pyrimidine‐2,6‐diamine scaffold (Figure S2, Supporting Information). The design of these compounds was detailed in a publication [52], where the authors aimed to develop dipyridamole analogues as potential equilibrative nucleoside transporter 1 (SLC29A1) inhibitors. Their study demonstrated that replacing the piperidine moiety with azocane led to improved bioactivity (Figure 7) It is noteworthy that this case study exemplifies the utilization of a macrocyclic moiety for drug modification, even though the primary scaffold being investigated is not inherently macrocyclic. Also, the authors of the original study highlighted that *“Further, not only does the ring size matter but also the ring flexibility is important”*, providing us with a testament to how specific macrocyclic features can be beneficial in a drug discovery project.

The SLC29 family encompasses four transporters, namely SLC29A1, SLC29A2, SLC29A3, and SLC29A4, with distinct roles in cellular transport processes. SLC29A1‐3 primarily mediates the transport of nucleobases, nucleosides, and nucleoside therapeutic analogs, while SLC29A4 is more prominently involved in the transport of biogenic amines, neurotoxins, and cationic therapeutics [53, 54]. These transporters exhibit wide expression patterns across various tissues, indicating their essential roles in mediating the uptake of nucleoside‐based drugs and endogenous nucleosides. Thus, the SLC29 family represents a significant target for the development of novel therapeutics in diverse therapeutic areas, including anticancer, antiviral, and antihypertensive drugs, among others [53, 54, 55].

In our workflow construction, we established a criterion for defining macrocycles as structures containing a minimum of nine atoms within the ring. Consequently, the presence of such structures in our dataset is to be expected, as for example, azonanes possess a ring consisting of exactly nine atoms. A similar observation can be made for the compounds associated with SLC10 (Sodium bile salt cotransport family). The SLC10 family consists of seven transporters, among which our dataset specifically includes records for SLC10A2. SLC10A2 is predominantly expressed in the intestine and plays a crucial role in the cholehepatic shunt. Dysfunction or deviations in the function of SLC10A2 have been associated with several conditions, including intrahepatic cholestasis, biliary atresia, and bile acid malabsorption [56, 57, 58, 59, 60].

The core of the recorded structure for SLC10A2 (Figure 5j) corresponds to benzothiepine rather than the 14‐membered heterocyclic ring (Figure S3, Supporting Information). The design and synthesis of the recorded compounds have been previously described in a publication [61] where the authors aimed to discover potent and nonsystemic inhibitors targeting the hepatic Sodium/Bile Acid Cotransporter (SLC10A2). While the inhibitory properties primarily originate from the benzothiepine moiety, the author demonstrated that the inclusion of the terminal 14‐membered heterocyclic ring, along with its linker, enhances the potency of the overall structure.

Interestingly, we found some compounds for which a macrocycle was not the main scaffold. Examples are the already mentioned SLC9 and SLC29 clusters (Figure 5e,f respectively). In these compounds macrocycles are typically sub‐fragments of a larger structure. Also, visual analysis with the t‐SNE map reveals their dissimilarity with typical macrocycles.

**Figure 8:**
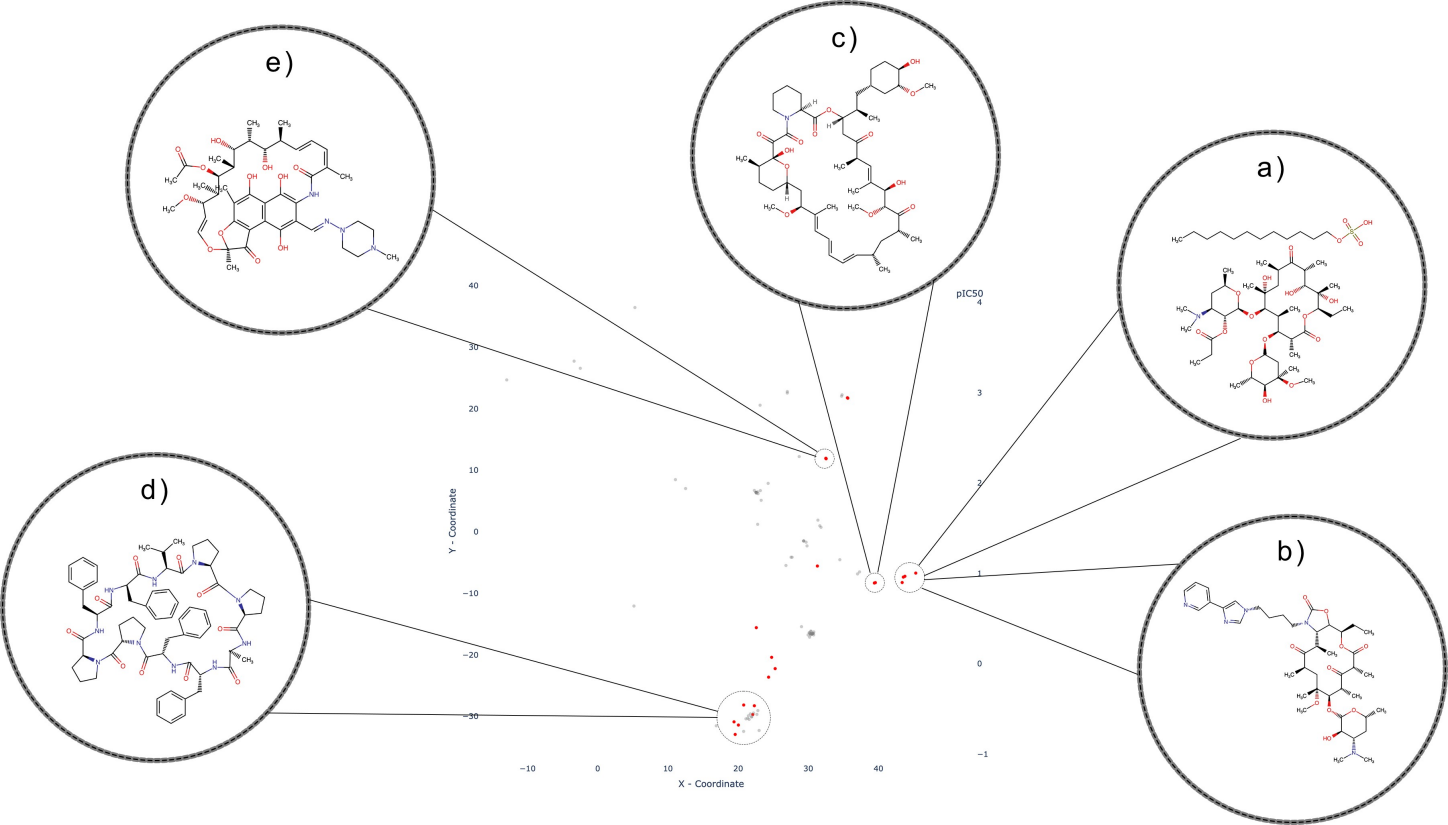
Distribution of SLCO (SLC21) Family with four clusters: Macrolide cluster shown with Erythromycin Estolate and Telithromycin as examples (a and b respectively); Macrolide cluster with Sirolimus as example (c); Cluster containing cyclic peptides with Antamanide as example (d); Polyketide cluster with Rifampin as an example (e).

In contrast to the aforementioned SLC families, where inhibitors tend to exhibit discernible clusters, the inhibitors belonging to the SLCO (Organic Anion Transporting Polypeptide, or SLC21) family demonstrate a different pattern. These inhibitors display either a widely dispersed distribution across the chemical space or form multiple scattered clusters, as depicted in Figure 8. Notably, a prominent cluster comprises macrolides, namely Erythromycin (Figure 8a) (here as salt Erythromycin Estolate), Clarithromycin, and Telithromycin (Figure 8b). Adjacent to this cluster, Sirolimus and its analogs form smaller clusters (Figure 8c). Within the SLCO family, other noteworthy inhibitors include peptide‐like macrocycles like Antamanide (Figure 8d), as well as various polyketides such as Rifampin and its analogs (Figure 8e). While these compounds do not exhibit a discrete cluster, they share a common origin as they are either natural products or derivatives. The SLCO family encompasses 11 transporters that play a pivotal role in regulating the uptake of drugs employed in antihypertensive therapy, such as Valsartan and Bosentan. Additionally, besides their remarkable polyspecificity [62], they are integral to the transport of bile acids, hormones, and other endogenous substrates [63].

**Figure 9:**
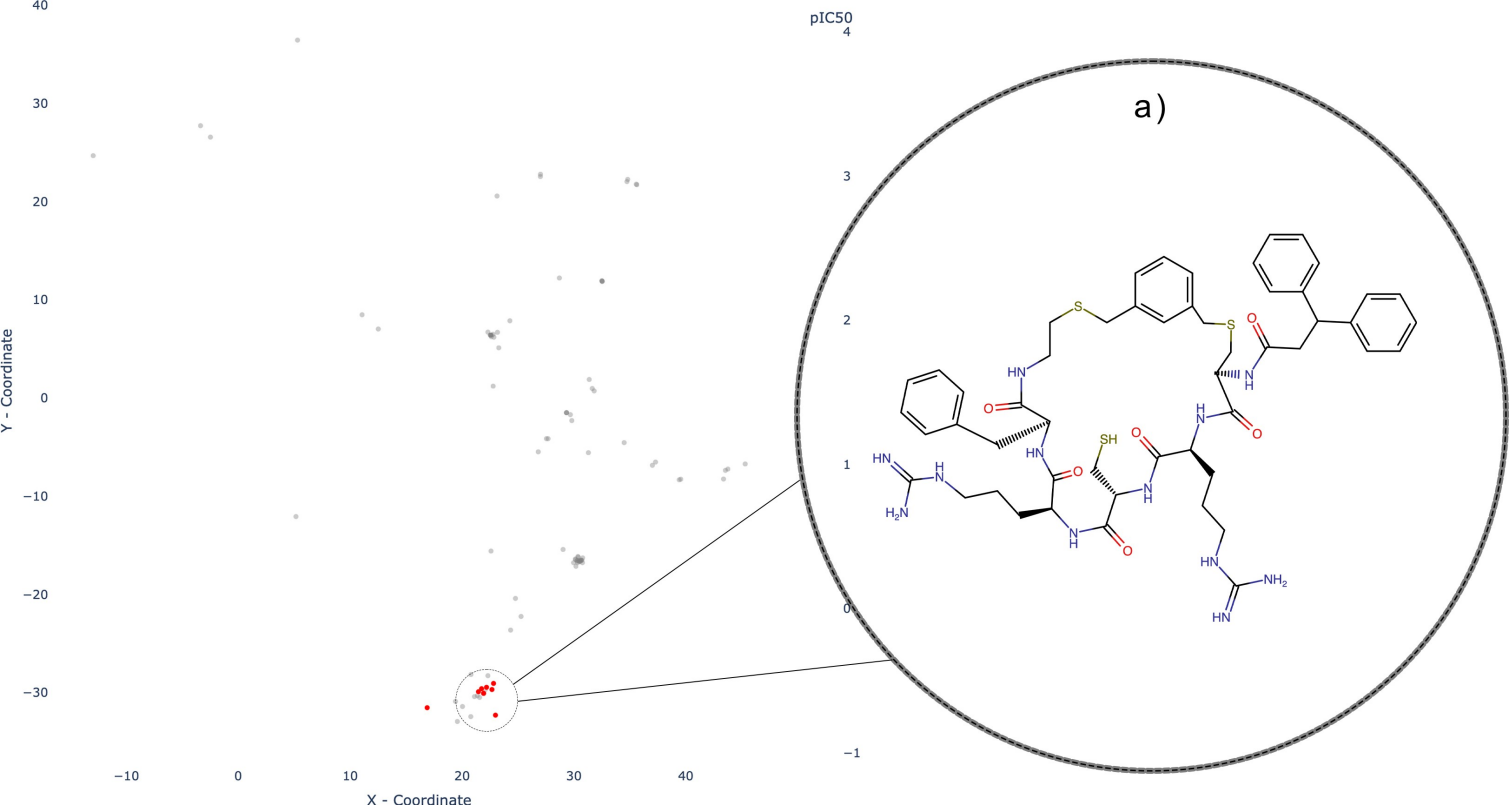
The cluster of SLC 40 family with mini Hepcidin analogue as an example.

The SLC40 family, also known as the Basolateral iron transporter family, has only one transporter, namely Ferroportin 1 (SLC40A1), which regulates the iron homeostasis [64]. It is prominently depicted as a well‐defined turquoise blue cluster in Figure 5. This cluster comprises 8 compounds (Figure 5g and 9) that exhibit a distinct and compact arrangement without any outliers. Notably, these compounds belong to the category of peptide‐like macrocycles (Figure 9a), specifically designed as mimetics of hepcidin, a regulator of iron homeostasis [65]. The compounds discussed in this study have been previously documented in a publication [65], wherein the authors employed cyclization as a strategic approach to minimize the utilization of uncommon or costlier amino acid derivatives. Through their investigation, they demonstrated the effectiveness and practicality of cyclization as a crucial synthetic step for the generation of novel hepcidin mimetics. Their findings highlight the utility of this cyclization methodology in the synthesis of structurally diverse compounds with potential therapeutic implications in the field of iron homeostasis regulation. Furthermore, this cluster is located in close vicinity to the SLC6 and SLCO families (shown in Figure 5), which also comprise peptide‐based compounds (Figure 11). The activities recorded for the compounds in this cluster display variations ranging from pEC50 6.22 to 7.66.

For the SLC65 family, specifically the Niemann‐Pick Transporter, only activity data for SLC65A1 are provided. This family encompasses two transporters that play a crucial role in cholesterol transport [66, 67].

**Figure 10:**
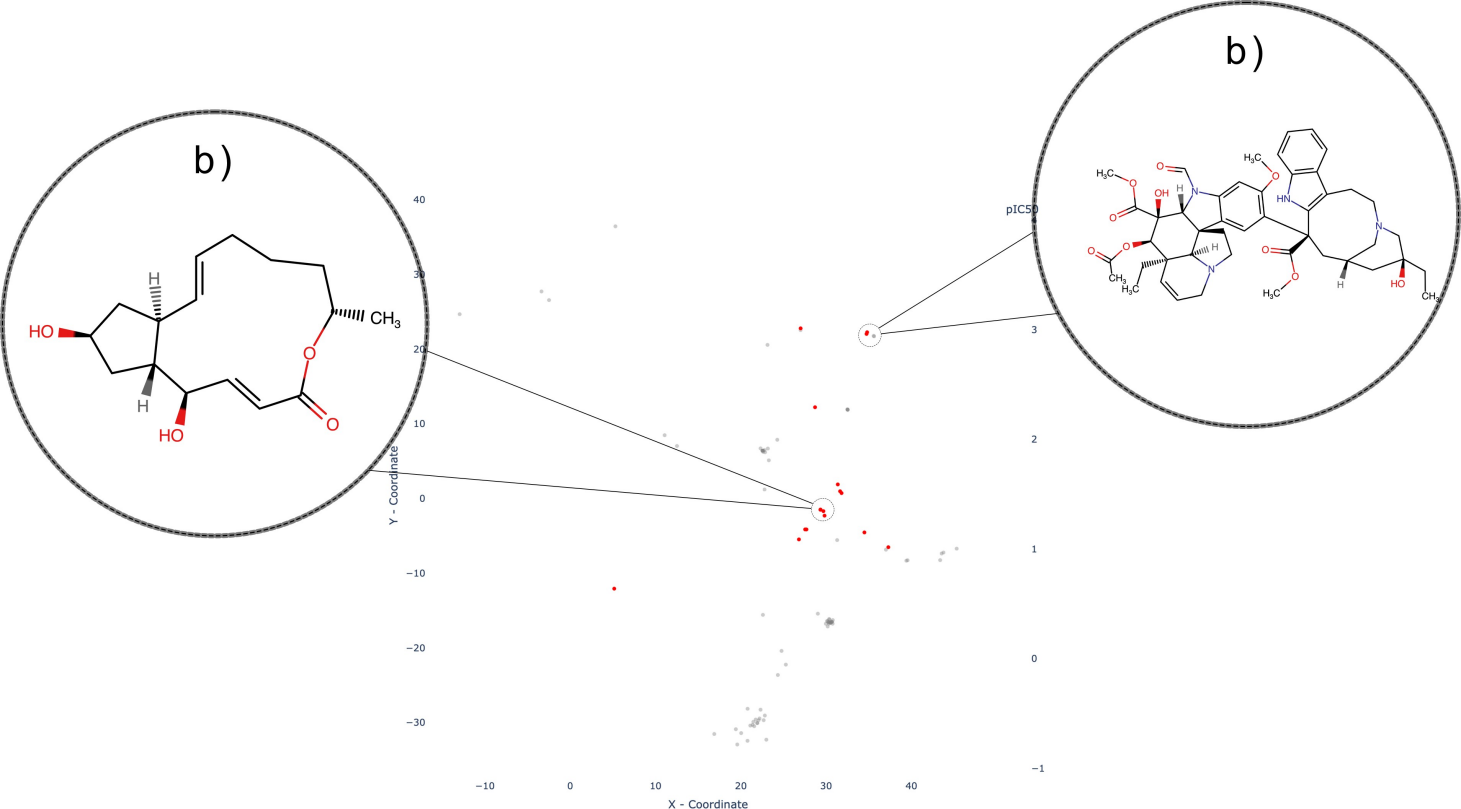
Distribution of SLC65 family with two shown clusters; Cluster containing Vincristine (a) and cluster containing macrocyclic esters (b).

The activity values obtained for this family are within a moderate range, spanning from 5.0 to 7.05. Similar to the compounds of the SLCO family, we observe a wide distribution of compounds across the chemical space, with only a few discernible clusters. This could indicate potential for varied structural motifs and functional characteristics among the compounds. One example of compounds exhibiting diverse structural motifs within the SLC65 family is a class of alkaloids derived from Catharanthus roseus, specifically Vinblastine and Vincristine (Figure 10a), which are known for their chemotherapeutic applications [68]. Additionally, we can also observe cluster of compounds including macrocyclic esters. (Figure 10b). Our visualizations demonstrate notable ligand promiscuity within two distinct families: SLCO and SLC65, as depicted in Figures 5h,i. Existing studies validate our finding of ligand promiscuity in SLCO transporters, such as SLCO1B1 [69]. However, our literature search did not reveal any previously documented instances of ligand promiscuity within the SLC65 family.

### “Familiarity” of macrocycles

By implementing our synonyms extraction protocol as described in the “Analysis of the Familiarity of Macrocycles” section of the Methods, and manual curation of results, we identified 22 macrocycles that satisfied the criteria for being considered well‐recognized compounds. Among these, the primary compound, Rifampicin, exhibited an impressive count of 193 synonyms. Table 1 provides the common names of the top 5 compounds. However, it can be inferred that the majority of macrocycles do not possess a high level of recognition or familiarity (see Figure 4).

**Table 1:** The top‐5 compounds by the number of synonyms extracted from PubChem.

Name	No. of synonyms	Type
Rifampicin	193	Ansamycins
Clarithromycin	188	Macrolides
Erythromycin	135	Macrolides
Cepharanthine	89	Alkaloids
Vinblastine	82	Alkaloids

In our analysis of compounds with fewer than five synonyms, we found only two–Demethylphalloin and Antamanide–with distinct individual names. Two others within the same category were referred to as ′Dipyridamole Analogue, 15′ and ′Dipyridamole Analogue, 16′. The remaining compounds were identified only by database identifiers and IUPAC names. In contrast, among the well‐recognized compounds, we could identify either common names or trade names for all but two, which were identified as ′CHEMBL1981103′ and ′CHEMBL1309093′.

### Two‐, three‐ and four‐ring macrocycles

**Figure 11:**
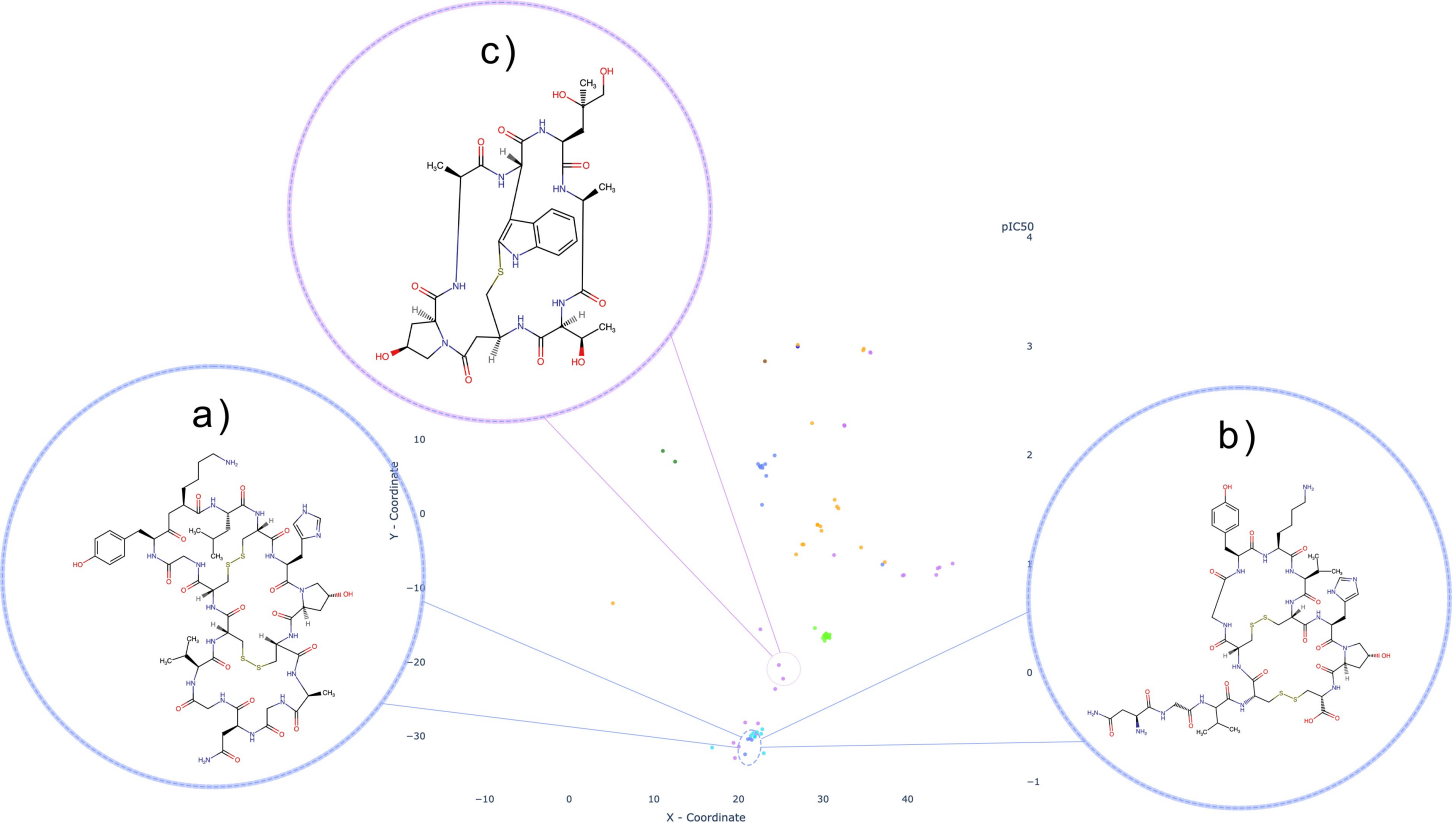
Representation of SLC6 family (corn blue) with two examples: three‐ring (a) and two‐ring macrocycle (b); SLCO (SLC21) family (purple) with Phalloidin, a two‐ring macrocycle. SLC40 family shown in light blue also comprises peptide‐like macrocycles.

**Figure 12:**
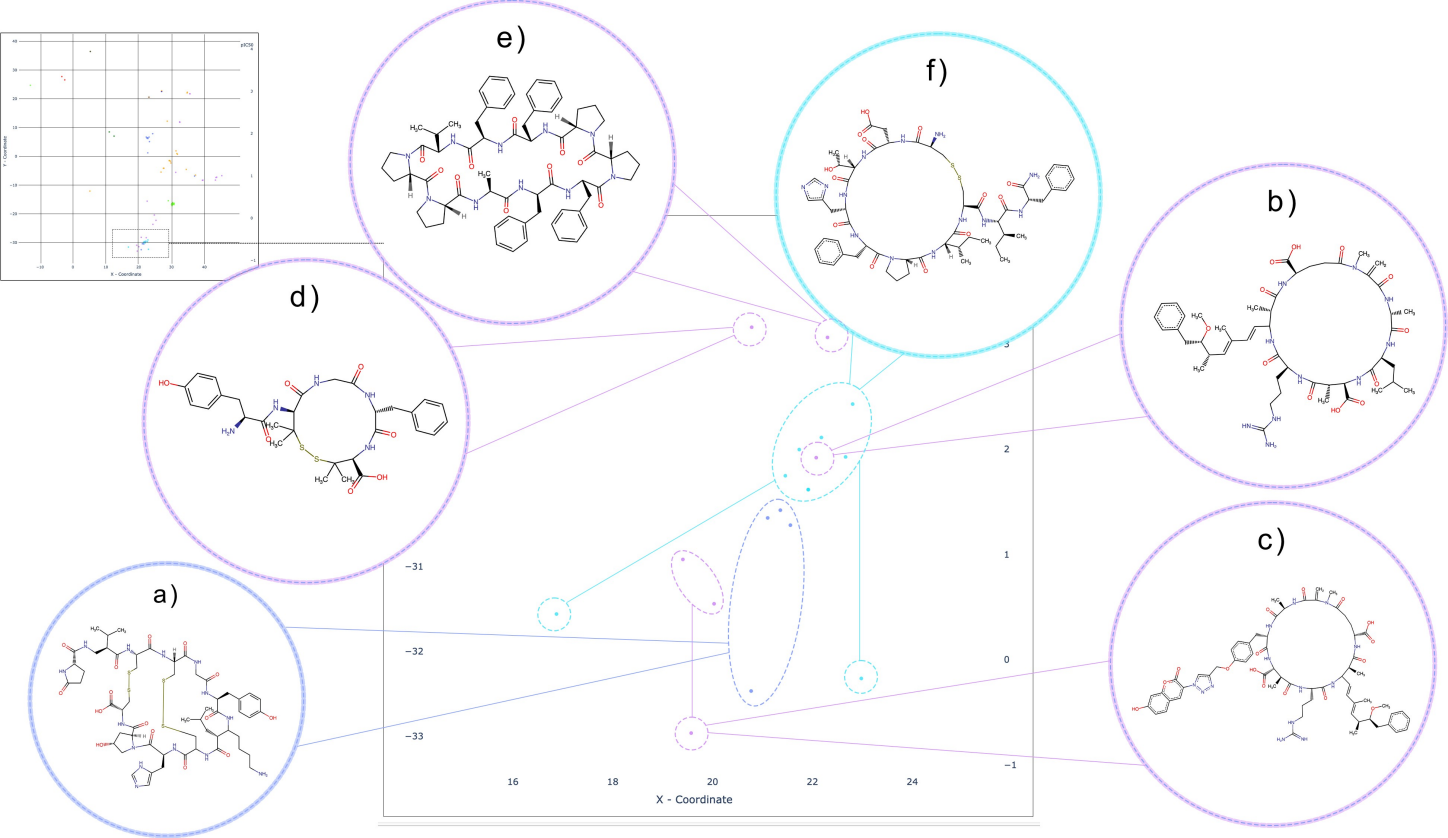
Detailed view on the “peptide‐like” cluster on the t‐SNE map: SLC6 family (corn‐blue) interacts with macrocycles which scaffold belong to the Conopeptide; an example shown in a) is a chi‐Conopeptide analogue; SLCO family (purple) interacts with macrocycles that comprise three different scaffolds; b) and c) both share the scaffold with Microcystin where b) is Microcystin LR and c) is it analogue; besides Microcystin, SLCO interacts with d) L‐Tyrosyl‐D‐penicillamyl‐glycyl‐L‐phenylalanyl‐D‐penicillamine (2‐>5)‐disulfide (DPDPE) and e) Antamanide; Tfhe macrocycles of SLC40 (turquoise blue) family shown in this cluster are all analogs of Hepcidin. One example is shown in f); (**NOTE**: Connection between cluster and a single point (as seen in Figures 12c,f), indicates that the entitles exhibit same scaffold type).

**Figure 13:**
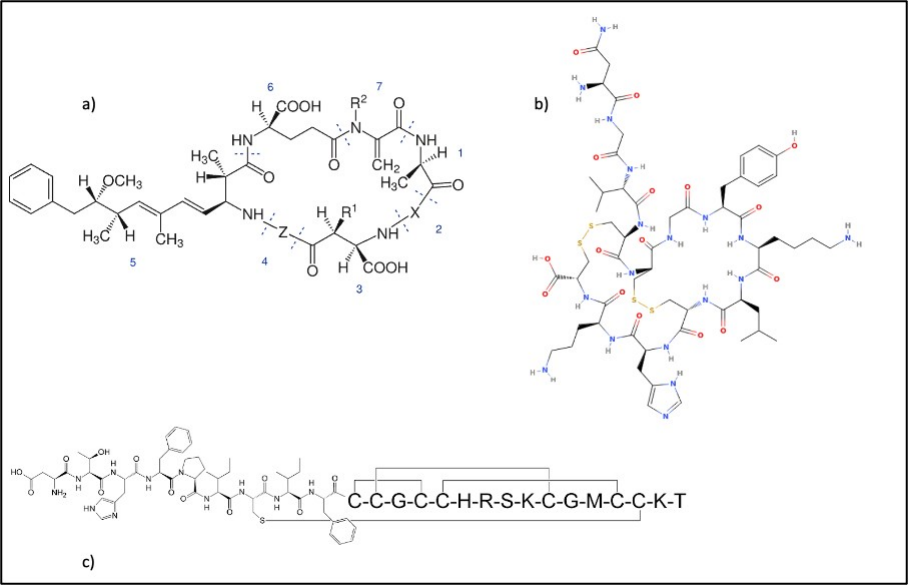
Representation of three out of five main scaffolds found in “peptide‐like” cluster shown in Figure 12: a) Main structure of Microcystin [75, 76]; b) Main structure of chi‐Conopeptide [77]; and c) main structure of the peptide Hormone Hepcidin [65] Another two scaffolds are: Antamanide and DPDPE (Figure 12e,d respectively).

As previously stated, we collected eight SLC‐macrocycles pairs whose macrocyclic core is either bicyclic, tricyclic, or even tetracyclic. All compounds have peptide‐like motives and among them we can also observe nature derived products such as Phalloidin (Figure 11c), a toxin used as an imaging tool [70, 71]. SLC families interacting with these compounds comprise SLC6 (Figure 11a,b) and SLCO which, together with the already discussed SLC40 family, form a well‐shaped cluster in the t‐SNE map. We conducted a visual inspection coupled with literature search of the structural framework of these compounds to identify shared characteristics that group multi‐ring macrocycles together with one‐ring macrocycles, beyond the presence of peptide‐like motives in the rings. Our analysis revealed five distinct scaffolds: Microcystin (Figure 13a), χ‐Conopeptide (Figure 13b), cyclic Hepcidin (Figure 13c), Antamanide, and DPDPE. The SLC6 family (corn‐blue) interacts in this cluster with two‐ and three ring cyclopeptides that are χ‐Conopeptide MrIA analoga (Figure 11a,b and 12a), researched for their potential to be used as analgesics [72, 73]. The SLCO family (purple) interacts with two different scaffolds. The first one is the already mentioned two‐ring macrocycle Phalloidin that lies outside the well‐defined “peptide‐like” cluster (Figure 11c), and another one is the heptapeptide Microcystin (Figure 13a), whose analogue (Figure 12b,c) lies within the “peptide‐like” cluster. Beside one‐ring Microcystin analogues, there is the previously mentioned decapeptide Antamanide (Figure 12e) and the opioid peptide [d‐penicillamine2,5]enkephalin (DPDPE) (Figure 12f) [74]. The four‐ring macrocycle that interacts with the transporter of the SLC40 family is also a Hepcidin (Figure 13c) analogue as the previously described one ring Hepcidin (Figure 12f) analogue. Despite the predominantly natural origin of these compound scaffolds, apart from the synthetic opioid DPDPE and the human liver‐produced hormone Hepcidin, we did not identify any other common features. Furthermore, no shared scaffold was observed between the afore mentioned three SLC families.

### Validation of the methodology using G‐protein coupled receptors (GPCRs)

As already mentioned, the created workflow is target class agnostic. To demonstrate that it is executable also for other target classes, we did a brief explanatory analysis for G‐coupled Receptors (GPCRs). GPCRs are a class of transmembrane receptors that play a crucial role in cell signaling. They are the largest family of membrane receptors, and are involved in a wide range of physiological processes, including regulation of the cardiovascular and nervous systems, sensation, and hormone signaling [78, 79, 80]. Due to the broad physiological roles of GPCRs and their significant importance in many diseases, it is not surprising that they have become one of the most important targets for drug discovery and development. Today, many successful drugs, such is Aripiprazole (known under trade name Abilify, which reached the top selling prescription drug in the US in 2014) and Trulicity (which was among the top 10 small molecule blockbuster drugs in the World in 2022) target GPCRs [79, 80]. The file GPCRTargets.xlxs (Supporting Information) containing identifiers for 411 GPCRs was downloaded from the website “IUPHAR/BPS Guide to PHARMACOLOGY” [81]. The search retrieved 811,098 bioactivity values, with macrocycles (range nine to 30) covering 2.5 % (20,532) of the ligand space. Comparing this outcome with the one for SLCs, it is evident that there are 24 times more data points regarding macrocycles interacting with GPCRs compared to SLCs. Applying the same filtering steps as for SLCs led to the numbers provided in Table 2.

**Table 2:** Data comparison between SLCs and GPCRs.

Target family	No. of retrieved data (ChEMBL)	No. of bioactivity values for macrocycles in ChEMBL (nine to 30)	No. of unique structures (based on InChi)	No. of unique compound–target pairs	No. of one‐ring macrocycles‐target pairs	No. of activity values <10 μM	No. of one‐ring macrocycle‐target pairs (IC50)
SLC	87,887	825	92	169	161	141	75
GPCR	811,098	20,532	3,543	7,444	7,206	7,013	2,228

The analysis of the results presented in Table 2 reveals remarkable distinctions between SLCs and GPCRs. Notably, there is a nine‐fold higher number of retrieved data from ChEMBL for GPCRs compared to SLCs. Additionally, GPCRs exhibit a much larger number of retrieved bioactivity values for macrocycles, which is 24 times higher within the ring size range of 9–30. However, when comparing the ratio of retrieved data to bioactivity values, a moderate difference is observed, with GPCRs showing a ratio of 2.5 % and SLCs exhibiting a lower ratio of 0.9 %.

Another significant comparison lies in the ratio of the number of unique structures (based on InChi) to the number of bioactivity values for macrocycles. In this regard, the ratio is 11.2 % for SLCs and 12.3 % for GPCRs, further highlighting the moderate difference between the two groups. Additionally, it is worth noting that one‐ring macrocycles account for 35.1 % of the total bioactivity values for macrocycles within the ChEMBL database for GPCRs, whereas SLCs comprise only 19.5 % of such occurrences. Moreover, the discrepancy between the number of one‐ring macrocycles associated with specific targets, for which IC50 values have been recorded, relative to the overall number of bioactivity values for macrocycles, is 3.0 % for SLCs and 10.9 % for GPCRs.

These findings support the assertion made in the introduction that SLCs remain an insufficiently explored target class [2, 5, 6]. In contrast, GPCRs have received significant scientific attention over several decades [78]. This discrepancy in research focus may explain the observed differences in the results presented in Table 2.

## CONCLUSION

The main goal of the work was to showcase the interaction landscape between SLCs and macrocycles through an automated and interactive workflow using KNIME. A web‐based tool, which is based on parametric t‐SNE, was created to visualize the interaction space of SLCs with macrocycles. The workflow has the ability to retrieve, analyze, and display data from the ChEMBL database.

Our workflow includes two interactive dashboards. The first dashboard displays the molecular properties of compounds as defined by the user, with the “Lipinski Rule of 5“ descriptors being used for demonstration purposes. The second dashboard shows the relationship between SLCs and the distribution of Ring Sizes, Activity Values, and the distinction between peptide‐like macrocycles and others. The limitation of the workflow lies in its inability to identify instances where a single compound has both IC50 and Ki values determined for the same target using the same assay, all reported in a single source. To provide high‐quality and consistent data to perform the analysis of the chemical space using t‐SNE, we had to remove those duplicates manually.

Using a parametric t‐SNE based chemical space visualization tool allowed to identify clusters of structurally similar compounds associated to distinct SLC families. Interestingly, in the case of SLC40, SLC6, and SLCO, compounds grouped together possess more than three peptide‐like motives within a macrocyclic ring, further demonstrating the discrimination ability of the parametric t‐SNE model.

The workflow can be used to study interactions between macrocycles and targets other than SLCs, as shown by an analysis for GPCRs. Our study indicates that the percentage of macrocycles functioning as GPCR ligands is greater than that of SLCs ligands. However, despite this, the overall prevalence of macrocycles in comparison to other molecules remains relatively low, not exceeding a few percent.

We are aware of the potential limitations of this analysis due to the known positive data bias in the ChEMBL dataset. Our prior study demonstrated that ChEMBL data is disproportionately biased towards active compounds, potentially overlooking inactive ones [82]. Given this, it's plausible that our analysis is similarly skewed towards active macrocycles.

In conclusion, the flexible approach we have proposed enables users to study the interaction landscape of macrocycles with a diverse range of target types, as we demonstrated with SLCs and GPCRs. It aids the user in gathering data from the ChEMBL database and processing it for further use. By using the workflow, scientists can quickly understand the current interaction landscape between macrocycles and a selected target class. Additionally, we showed that the parametric t‐SNE approach can be a useful tool to explore the chemical space of macrocycles.

## SUPPORTING INFORMATION

Additional supporting information can be found online in the Supporting Information section at the end of this article.

## Conflict of interests

The authors declared that they have no conflict of interest.

1

## Supporting information

As a service to our authors and readers, this journal provides supporting information supplied by the authors. Such materials are peer reviewed and may be re‐organized for online delivery, but are not copy‐edited or typeset. Technical support issues arising from supporting information (other than missing files) should be addressed to the authors.

Supporting Information

## Data Availability

The data that support the findings of this study are openly available in Phaidra at https://doi.org/10.25365/phaidra.447.
