# Supplementary material for: The macrocycle inhibitor landscape of SLC‐transporter
Source: Mol Inform. 2024 Mar 5;43(5):e202300287. doi: 10.1002/minf.202300287 (PMC11475418; doi:10.1002/minf.202300287)
Supplement: Supplementary file 1 — Supporting Information [file MINF-43-e202300287-s001.pdf]

molecular informatics

Supporting Information

The macrocycle inhibitor landscape of SLC-transporter

Nejra Granulo[1, 2] | Sergey Sosnin[1] | Daniela Digles[1] | Gerhard F. Ecker[1, 2]

Supporting Information for the manuscript: The macrocycle inhibitor landscape of SLC-transporter:

Nejra Granulo [1,2], Sergey Sosnin [1], Daniela Digles [1], Gerhard F. Ecker* [1,2]

[1] University of Vienna, Department of Pharmaceutical Sciences, Josef Holaubek Platz 2, 1090 Wien, Austria

[2] University of Vienna, Research Platform NeGeMac - Next Generation Macrocycles to Address Challenging Protein Interfaces, 1090 Vienna, Austria

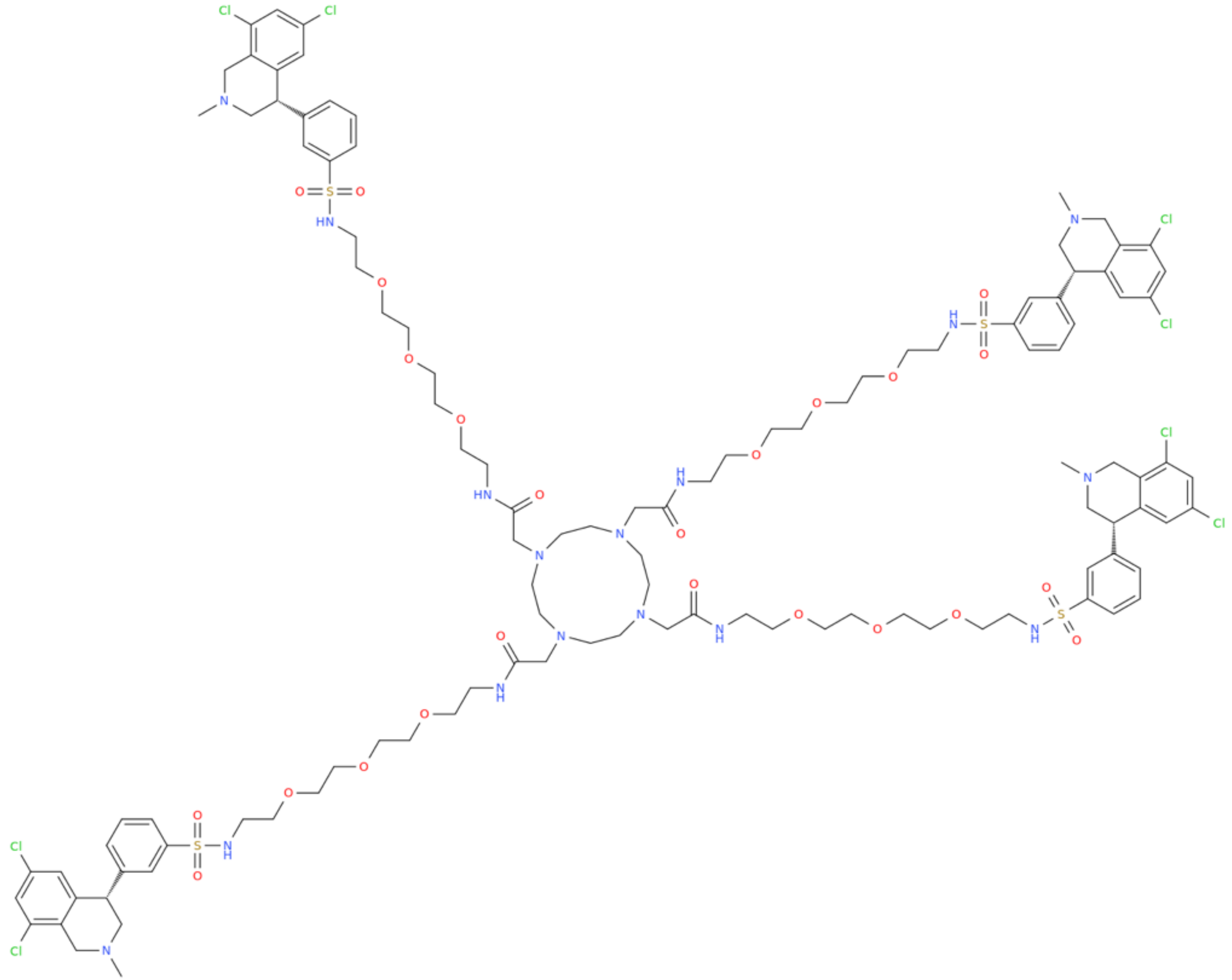

Figure 1: SLC9 scaffold.

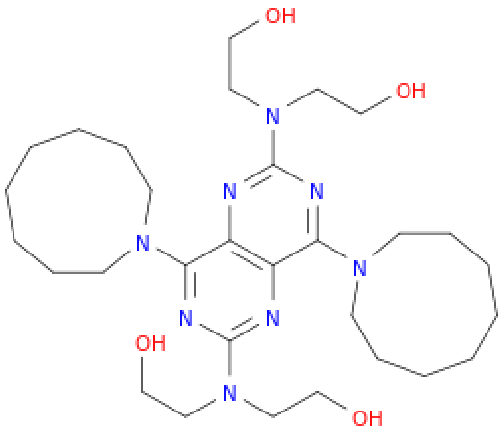

Figure 2: SLC29 scaffold.

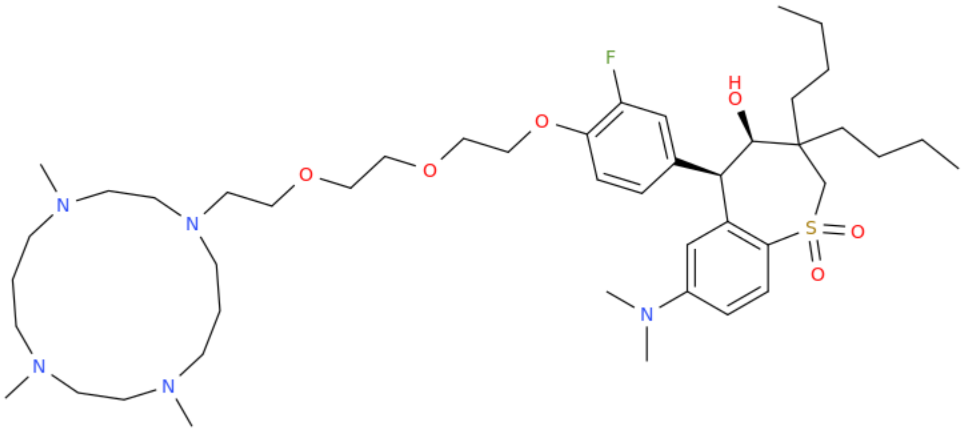

Figure 3: SLC10 scaffold.

Table 1: Distribution of SLC Transporter per ring size with unique count

RingSize	Unique count (SLC name)	Unique concatenate (SLC name)
**10**	7	SLC65A1, SLC5A2, SLC6A2, SLC6A3, SLC6A4, SLCO1B1, SLCO1B3
**11**	5	SLC65A1, SLC5A2, SLC5A7, SLCO1B1, SLCO1B3
**12**	6	SLC65A1, SLC25A5, SLC25A6, SLC34A2, SLC5A2, SLC9A3
**12, 14, 16**	3	SLC6A2, SLC6A3, SLC6A4
**12, 16**	6	SLC22A1, SLC6A2, SLC6A3, SLC6A4, SLCO1B1, SLCO1B3
**13**	3	SLC65A1, SLC5A2, SLC5A7
**14**	18	SLC65A1, SLC10A2, SLC22A1, SLC22A7, SLC25A5, SLC25A6, SLC40A1, SLC47A1, SLC5A2, SLC5A4, SLC6A2, SLC6A3, SLC6A4, SLCO1A2, SLCO1B1, SLCO1B3, SLCO1C1, SLCO2B1
**14, 17**	3	SLCO1B1, SLCO1B3, SLCO2B1
**14, 28, 29, 30**	1	SLC40A1
**15**	15	SLC22A6, SLC22A7, SLC25A5, SLC25A6, SLC5A1, SLC5A2, SLC5A4, SLC5A7, SLC6A2, SLC6A3, SLC6A4, SLCO1A2, SLCO1B1, SLCO1B3, SLCO1C1
**15, 18**	3	SLCO1B1, SLCO1B3, SLCO2B1
**16**	10	SLC65A1, SLC22A5, SLC5A1, SLC5A2, SLC6A2, SLC6A3, SLC6A4, SLCO1B1, SLCO1B3, SLCO2B1
**17**	3	SLC6A2, SLC6A3, SLC6A4
**18**	13	SLC65A1, SLC17A5, SLC22A2, SLC25A5, SLC25A6, SLC2A1, SLC47A1, SLC47A2, SLC6A2, SLC6A3, SLC6A4, SLCO1B1, SLCO1B3
**19**	3	SLC6A2, SLC6A3, SLC6A4
**20**	5	SLC6A2, SLC6A3, SLC6A4, SLCO1B1, SLCO1B3
**20, 22**	2	SLC6A2, SLC6A3
**20, 22, 23**	1	SLC6A2

**21**	8	SLC10A1, SLC22A1, SLC22A6, SLC6A2, SLC6A3, SLC6A4, SLCO1B1, SLCO1B3
**21, 22**	1	SLC6A2
**21, 24**	1	SLC6A2
**22**	2	SLCO1B1, SLCO1B3
**22, 23**	1	SLC6A2
**23**	5	SLC6A2, SLC6A3, SLC6A4, SLCO1B1, SLCO1B3
**24**	10	SLC22A1, SLC2A1, SLC2A4, SLC6A2, SLC6A3, SLC6A4, SLCO1A2, SLCO1B1, SLCO1B3, SLCO2B1
**25**	8	SLC40A1, SLC6A2, SLC6A3, SLC6A4, SLCO1A2, SLCO1B1, SLCO1B3, SLCO2B1
**26**	3	SLC40A1, SLCO1B1, SLCO1B3
**28**	2	SLCO1B1, SLCO1B3
**29**	6	SLC25A5, SLC25A6, SLC6A2, SLC6A3, SLC6A4, SLCO1B1
**30**	5	SLC40A1, SLC6A2, SLC6A3, SLC6A4, SLCO1B1
**9**	9	SLC65A1, SLC22A1, SLC29A1, SLC6A2, SLC6A3, SLC6A4, SLCO1B1, SLCO1B3, SLCO2B1

Table 2: Distribution of Ring Sizes per SLCs with unique count

SLC name	Unique count (RingSize)	Unique concatenate(RingSize)
**SLC10A1**	1	21
**SLC10A2**	1	14
**SLC17A5**	1	18
**SLC22A1**	5	21, 9, 14, 24; 3 RINGS: 12, 16, 16
**SLC22A2**	1	18
**SLC22A5**	1	2 RINGS: 16, 16
**SLC22A6**	2	15, 21
**SLC22A7**	2	14, 15
**SLC25A5**	5	12, 15, 18, 14, 29
**SLC25A6**	5	12, 15, 18, 14, 29
**SLC29A1**	1	9
**SLC2A1**	2	24, 18
**SLC2A4**	1	24
**SLC34A2**	1	12
**SLC40A1**	5	14, 25, 26, 30; 4 RINGS: 14, 28, 29, 30
**SLC47A1**	2	18, 14
**SLC47A2**	1	18
**SLC5A1**	2	15, 16
**SLC5A2**	7	13, 12, 10, 14, 11, 15, 16

**SLC5A4**	2	14, 15
**SLC5A7**	3	13, 11, 15
**SLC65A1**	8	18, 13, 12, 9, 14, 10, 11, 16
**SLC6A2**	22	9, 10, 14, 15, 16, 17, 18, 19, 20, 21, 23, 24, 25, 29, 30; 2 RINGS: 22, 23; 21, 24; 21, 22; 20, 22; 12, 16; 3 RINGS: 20, 22, 23; 12, 14, 16
**SLC6A3**	18	9, 10, 14, 15, 16, 17, 18, 19, 20, 21, 23, 24, 25, 29, 30; 2 RINGS: 20, 22; 12, 16; 3 RINGS: 12, 14, 16
**SLC6A4**	17	9, 10, 14, 15, 16, 17, 18, 19, 20, 21, 23, 24, 25, 29, 30; 2 RINGS: 12, 16; 3 RINGS: 12, 14, 16
**SLC9A3**	1	12
**SLCO1A2**	4	14, 15, 24, 25
**SLCO1B1**	20	9, 10, 11, 14, 15, 16, 18, 20, 21, 22, 23, 24, 25, 26, 28, 29, 30; 2 RINGS: 15, 18; 12, 16; 14, 17;
**SLCO1B3**	18	9, 10, 11, 14, 15, 16, 18, 20, 21, 22, 23, 24, 25, 26, 28; 2 RINGS: 15, 18; 12, 16; 14, 17;
**SLCO1C1**	2	14, 15
**SLCO2B1**	7	9, 14,16, 24, 25; 2 RINGS: 14, 17; 15,18
